# A Survey Assessment of Nuclear Medicine Trainees’ Participation and Impact in Multidisciplinary Cancer Conferences: A Single-Center Study

**DOI:** 10.7759/cureus.92014

**Published:** 2025-09-10

**Authors:** Ghazal Norouzi, Bilquis Hyder Ali, Farzad Abbaspour, Eugene Leung, Alireza Khatami

**Affiliations:** 1 Division of Nuclear Medicine and Molecular Imaging, Department of Medicine, The Ottawa Hospital, University of Ottawa, Ottawa, CAN; 2 Division of Nuclear Medicine and Molecular Imaging, Department of Medicine, McGill University, Montreal, CAN

**Keywords:** interprofessional collaboration, medical education, multidisciplinary cancer conferences (mccs), nuclear medicine trainees, patient outcome

## Abstract

Background: Multidisciplinary cancer conferences (MCCs) are essential forums for collaborative oncology decision-making. However, existing literature has primarily examined the role of attending specialists and has rarely differentiated effects by specialty. The contributions of trainees, particularly in nuclear medicine, have been largely overlooked, leaving a gap in understanding how their participation influences both educational outcomes and patient management. This study addresses this gap by systematically evaluating the perceived impact of nuclear medicine trainees in MCCs.

Methods: A cross-sectional survey was distributed to 73 healthcare professionals at a tertiary medical center, including nuclear medicine specialists, trainees, and clinicians from surgery, oncology, and radiology. The survey included Likert-scale and multiple-choice questions to assess perceptions of trainee contributions to interprofessional collaboration, clinical decision-making, and patient outcomes. Descriptive statistics were calculated, and analysis of variance (ANOVA) and chi-square tests were applied to analyze Likert-scale responses and compare responses between nuclear medicine and non-nuclear medicine specialists. P-values < 0.05 were considered statistically significant.

Results: Of the 73 respondents, 57 (78.1%) indicated that nuclear medicine trainees enhanced interprofessional collaboration, while 56 (76.7%) reported a positive influence on patient care. Additionally, 60 (82.2%) perceived an educational benefit through enriched clinical knowledge. Chi-square analysis revealed no significant differences in perceptions across professional groups (p = 0.568). Reported barriers included inconsistent attendance, limited clinical experience, and time constraints.

Conclusion: Nuclear medicine trainees play a valuable role in MCCs by enriching clinical discussions, supporting patient care, and contributing to professional development. To maximize their impact, structured learning opportunities, increased mentorship, and improved logistical support are recommended. These findings emphasize the importance of formally integrating trainees into MCC workflows to enhance both educational and clinical outcomes.

## Introduction

Multidisciplinary cancer conferences (MCCs), also known as multidisciplinary tumor boards (MTBs), are essential forums where clinicians from diverse specialties collaborate to review complex oncology cases and develop comprehensive treatment plans. By integrating perspectives from medical oncologists, hematologists, surgeons, radiologists, nuclear medicine specialists, pathologists, radiation oncologists, and palliative care providers, MCCs ensure patient-centered decision-making and often determine eligibility for clinical trials [1-6].

Beyond patient management, MCCs also serve as valuable educational settings, offering trainees exposure to real-world interprofessional decision-making [7-10]. Nuclear medicine specialists play a particularly important role through the use of advanced imaging modalities such as positron emission tomography (PET) and single-photon emission computed tomography (SPECT), which are critical for diagnosis, staging, and monitoring [7]. However, despite the centrality of imaging in oncology, the contributions of nuclear medicine trainees (residents and fellows who prepare cases with supervising staff and present findings at MCCs) remain underexplored. To our knowledge, no prior study has systematically examined how trainees, across any specialty, influence interprofessional collaboration, patient care, or professional development within these conferences. This represents an important knowledge gap.

Existing literature highlights barriers to meaningful trainee involvement, including limited clinical experience, logistical constraints, and competing service duties [11-13]. What has not been addressed is whether structured curricular approaches can overcome these challenges and enhance the value of trainee participation. To address this, the nuclear medicine training program at the University of Ottawa introduced a dedicated rotation assigning trainees to MCC blocks. This structure ensures consistent attendance, supervised case presentations, and ongoing trainee involvement across all weekly MCCs. The present study evaluates the impact of this curricular innovation by systematically assessing nuclear medicine trainees’ contributions to interprofessional collaboration, their perceived influence on patient outcomes, and their role in professional development across specialties.

## Materials and methods

This study was approved by the institutional ethics committee. A cross-sectional survey was conducted to evaluate the perceived value and impact of nuclear medicine trainees in MCCs at a tertiary medical center. The anonymous survey was developed by the research team based on a literature review and adaptation of previously published instruments. The survey included six multiple-choice questions, five Likert-type questions, and two open-ended free-text questions, covering participant expertise, MCC participation, contributions to discussions, and perceived influence on rounds and patient outcomes. Free-text responses were analyzed thematically to identify recurring patterns, barriers, and actionable recommendations. All MCC participants were invited via email; participation was voluntary. Seventy-three participants completed the survey, including 37 nuclear medicine specialists and trainees and 36 clinicians from surgery, medical oncology, radiation oncology, radiology, urology, hematology, pathology, gastroenterology, pediatrics, and nursing.

Quantitative responses were analyzed with descriptive statistics. Comparisons between nuclear medicine and non-nuclear medicine respondents were made using analysis of variance (ANOVA) for Likert-scale items and Chi-square tests for categorical variables, chosen to assess differences in perceptions and frequencies. Assumptions of normality and homogeneity of variances were evaluated, and missing data (<5%) were handled by pairwise deletion. All analyses were conducted in MS Excel (Microsoft Corporation, Redmond, Washington, United States).

## Results

Demographics and participation

The survey included 73 participants representing a wide range of specialties. Approximately half of the respondents were from the field of nuclear medicine, including both specialists and trainees, while the remaining participants represented a diverse mix of other specialties such as surgery, oncology, pathology, pediatrics, and additional fields, including radiology, urology, endocrinology, and gastroenterology. The level of clinical experience among participants ranged from less than one year to over a decade, reflecting a broad spectrum of professional backgrounds.

Participants reported involvement in a wide range of MCC types, with the most common being lymphoma/PET, thoracic, and hepatobiliary rounds. Other rounds, such as GI, melanoma, GU, pediatrics, breast, endocrine, and foregut, were also well represented. One respondent reported attending multiple rounds in their role as a pathologist. In terms of attendance frequency, the majority of respondents indicated weekly participation in MCCs, followed by monthly, daily, and rare attendance.

Among non-nuclear medicine specialists, most identified their primary role as contributing to interdisciplinary discussions and treatment planning, with a smaller portion serving as the referring physicians for cases presented at MCCs. A detailed breakdown of the participants' specialties, experience levels, MCC involvement, and roles is provided in Table 1.

**Table 1:** Summarized demographics and participation characteristics of respondents NM: nuclear medicine; MCC: multidisciplinary cancer conference; PET: positron emission tomography

Category	Subcategory	n (% out of 73)
Specialty	Nuclear medicine specialists; trainees	28 (38.3%); 9 (12.3%)
	General/colorectal surgery	9 (12.3%)
	Hematology	6 (8.2%)
	Medical oncology; radiation oncology	4 (5.5%) each
	Pathology; pediatrics; non-NM trainees; nursing	2 (2.7%) each
	Thoracic surgery; radiology; urology; endocrinology; gastroenterology	1 (1.4%) each
Years of experience	0-1 year	9 (12.3%)
	2-5 years	22 (30.1%)
	6-10 years	18 (24.7%)
	>11 years	24 (32.9%)
MCC round type	Lymphoma/PET	13 (17.8%)
	Thoracic	11 (15.1%)
	Hepatobiliary	10 (13.7%)
	Gastrointestinal (GI)	9 (12.3%)
	Melanoma	6 (8.2%)
	Genitourinary (GU); pediatrics; breast; endocrine	5 (6.8%) each
	Foregut	3 (4.1%)
	Attended >1 round as pathologist	1 (1.4%)
MCC attendance frequency	Daily; rarely	6 (8.2%) each
	Weekly	45 (61.7%)
	Monthly	16 (21.9%)
Non-nuclear medicine respondents' role	Contributed to discussion/treatment planning	23/36 (63.9%)
	Referred cases	9/36 (25%)
	Other/unspecified	4/36 (11.1%)
n: number of participants/responses; %: percentage; total participants/responses: 73; *non-nuclear medicine participants/responses: 36		

A series of questions was designed using Likert scales to evaluate the impact, significance, and effectiveness of nuclear medicine trainees’ participation in MCCs, with the results as follows:

Impact of Trainees’ Participation on Interprofessional Collaboration

**Figure 1:**
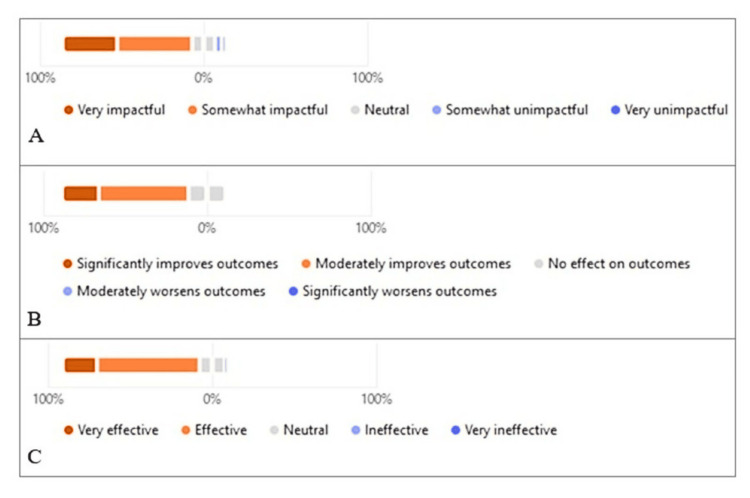
Likert scale responses (A) Impact of trainees’ participation on interprofessional collaboration. (B) Significance of trainees’ participation in MCC rounds on patient outcome. (C) Effectiveness of trainees’ participation in enhancing clinical skills and knowledge

The involvement of nuclear medicine trainees in MCCs was widely perceived as beneficial for interprofessional collaboration. Of the respondents, 24/73 (32.8%) considered the trainees' participation to be very impactful, 33/73 (45.2%) viewed it as somewhat impactful, and 10/73 (13.7%) rated the impact as neutral. Only 5/73 (6.8%) responded negatively, with 3/73 (4.1%) rating the participation as somewhat unimpactful and 2/73 (2.7%) as very unimpactful. These results highlight the overall positive perception of nuclear medicine trainees’ role in fostering collaboration between different specialties. Responses from non-nuclear medicine specialists followed a similar trend: 12/36 (33.3%) rated the participation as very impactful, 14/36 (38.9%) as somewhat impactful, 6/36 (16.7%) as neutral, and 3/36 (8.3%) as somewhat unimpactful. No respondents in this group rated the participation as very unimpactful. Figure 1A illustrates the Likert scale responses to this question.

Significance of Trainees’ Participation in MCC Rounds on Patient Outcomes

Regarding patient outcomes, all 73 respondents rated their perceptions of nuclear medicine trainees’ involvement in MCC rounds using a Likert scale. Of these, 16/73 (21.9%) believed that the trainees' involvement significantly improved outcomes, 39/73 (53.4%) felt that it moderately improved outcomes, and 16/73 (21.9%) reported no effect. Notably, no respondents indicated that the involvement had a negative impact on patient outcomes. Responses from the non-nuclear medicine specialists followed a similar trend: 6/36 (16.7%) rated the impact as significantly improving outcomes, 19/36 (52.8%) as moderately improving, and 11/36 (30.6%) as having no effect. Again, no responses in this cohort suggested any worsening of patient outcomes. These findings suggest that the contributions of nuclear medicine trainees are viewed as beneficial to the management of complex cancer cases, particularly in contexts where advanced imaging plays a critical role. Figure 1B presents the detailed Likert scale responses from all participants.

Effectiveness of Participation

The majority of respondents, 60/73 (82.2%), rated the effectiveness of nuclear medicine trainees' participation in multidisciplinary rounds in enhancing their clinical skills and knowledge as either very effective (15/73, 20.5%) or effective (45/73, 61.6%). A neutral effect was reported by 11/73 (15.1%), while only 1/73 (1.4%) described the experience as very ineffective. The distribution and trend of responses among non-nuclear medicine specialists were similar, with 5/36 (13.9%) rating the impact as very effective, 22/36 (61.1%) as effective, and 9/36 (25.0%) as neutral. No responses in this group indicated the trainees’ participation was ineffective. These results affirm that nuclear medicine trainees are considered an asset to the learning environment during MCCs, contributing to both their own clinical development and that of other healthcare professionals. Figure 1C presents the Likert scale responses to this question.

**Figure 2:**
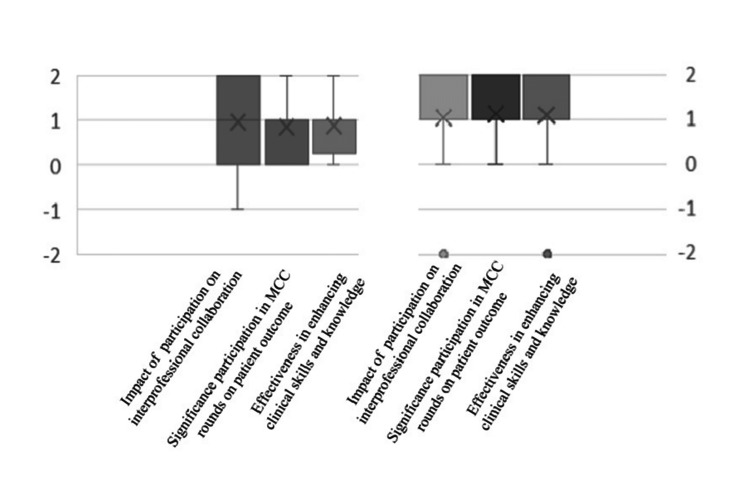
The impact, significance, and effectiveness of nuclear medicine participation in MCCs between two groups 2 = very positive; 1 = positive; 0 = no positive or negative; -1 = negative; -2 = very negative

The results of the ANOVA single-factor analysis demonstrate that there is no statistical difference in the positive impact of trainees’ participation in MCCs between nuclear medicine and non-nuclear medicine specialists (p-value = 0.568). Figure 2 shows the impact, significance, and effectiveness of nuclear medicine participation in MCCs based on the Likert questionnaire between the two groups.

Contributions of Nuclear Medicine Trainees

When asked about the specific contributions of nuclear medicine trainees, the most frequent responses were as follows: strengthening interactions between trainees and faculty (49/73, 67.2%), enhanced understanding of nuclear medicine imaging (43/73, 58.9%), enhanced communication between disciplines (39/73, 53.4%), improved collaboration with other specialties (37/73, 50.7%), and support for professional development (35/73, 47.9%). Other valuable contributions mentioned were better understanding of complex imaging techniques (33/73, 45.2%), improved patient management (31/73, 42.5%), providing fresh perspectives on cases (23/73, 31.5%), help in accelerating patient management (21/73, 28.8%), improved team dynamics (20/73, 27.4%), and bridging the gap between theory and practice (19/73, 26.0%). These contributions underscore the educational value that nuclear medicine trainees bring to MCCs, not only for their own learning but also in enriching the collaborative dynamics among specialists from various fields. The evaluation of responses from non-nuclear medicine specialists showed no statistical significance compared to the overall responses (p-value = 0.346), showing the same top two contributions, though with lower percentages. Fresh perspectives on cases were more appreciated among this cohort and stood in third place. Improved team dynamics, accelerated management, and bridging the gap between theory and practice remained at the bottom of the list (Table 2).

**Table 2:** Respondents opinion about specific contributions that nuclear medicine trainees bring to multidisciplinary rounds

Specific contribution of nuclear medicine trainees to MCC rounds	n (%) among 73 responses in all cohort	n (%) among 36 responses non- nuclear medicine experts	p-value
Strengthened interaction between trainees and faculty	49/73 (67.2%)	23/36 (63.9%)	0.737
Enhanced understanding of nuclear medicine imaging	43/73 (58.9%)	16/36 (44.4%)	0.154
Enhanced communication between disciplines	39/73 (53.4%)	15/36 (41.7%)	0.248
Improved collaboration with other specialties	37/73 (50.7%)	12/36 (33.3%)	0.087
Support for professional development	35/73 (47.9%)	15/36 (41.7%)	0.536
Better understanding of complex imaging techniques	33/73 (45.2%)	13/36 (36.1%)	0.366
Improved patient management strategies	31/73 (42.5%)	10/36 (27.8%)	0.137
Fresh perspectives on cases	23/73 (31.5%)	16/36 (44.4%)	0.185
Accelerated management by facilitating further imaging	21/73 (28.8%)	6/36 (16.7%)	0.169
Improved team dynamics	20/73 (27.4%)	9/36 (25.0%)	0.790
Bridging the gap between theory and practice	19/73 (26.0%)	7/36 (19.4%)	0.448

Contribution of Attending the Round to the Education of Nuclear Medicine Trainees

The majority of respondents agreed that participation builds confidence in trainees (62/73, 84.9%), encourages critical thinking (56/73, 76.7%), fosters communication skills (54/73, 74.0%), provides real-time learning opportunities, facilitates active learning for trainees, and engages trainees with faculty members-each receiving similar attention at 51/73 (69.9%), and enhances teamwork abilities (49/73, 67.1%). Offering opportunities to receive critical feedback was rated the lowest at 25/73 (34.2%). Among non-nuclear medicine specialists, fostering communication skills and providing real-time learning opportunities were top-rated, each with 31/36 (86.1%), followed by encouraging clinical thinking at 30/36 (83.3%), building confidence at 29/36 (80.6%), facilitating active learning for trainees at 29/36 (80.6%), providing engagement with faculty at 27/36 (75.0%), enhancing teamwork abilities at 26/36 (72.2%), and offering opportunities to receive critical feedback, which ranked lowest at 13/36 (36.1%). There was no statistical difference between the opinions of the non-nuclear medicine subgroup and the whole cohort on this matter (p-value = 0.409). Table 3 summarizes the abovementioned results.

**Table 3:** Contribution of participation in multidisciplinary rounds to the education of nuclear medicine trainees

Contribution of attending rounds to the education of nuclear medicine trainees	n (%) among 73 responses in all cohort	n (%) among 36 responses non- nuclear medicine experts	p-value
Builds confidence	62/73 (84.9%)	29/36 (80.6%)	0.563
Encourages critical thinking	56/73 (76.7%)	30/36 (83.3%)	0.426
Fosters communication skills	54/73 (74.0%)	31/36 (86.1%)	0.150
Provides real-time learning opportunities	51/73 (69.9%)	31/36 (86.1%)	0.065
Facilitates active learning for trainees	51/73 (69.9%)	29/36 (80.6%)	0.235
Provides engagement with faculty members	51/73 (69.9%)	27/36 (75.0%)	0.576
Enhances teamwork abilities	49/73 (67.1%)	26/36 (72.2%)	0.589
Offers opportunities to receive critical feedback	25/73 (34.2%)	13/36 (36.1%)	0.848
n=Number of responses. %=Percentage			

Challenges Encountered

Several challenges were identified regarding the participation of nuclear medicine trainees in MCCs that might hinder their ability to contribute fully. The most common challenges included limited understanding of other specialties (24/73, 32.9%), time constraints (22/73, 30.1%), limited clinical experience (19/73, 26.0%), lack of confidence in presenting or participating (16/73, 21.9%), and insufficient involvement in case discussions (12/73, 16.4%). The least commonly reported challenges were lack of familiarity with the cases being discussed and difficulty in explaining complex nuclear medicine concepts, both cited by 10/73 (13.7%). Among non-nuclear medicine specialists, the ranking and frequency of reported challenges differed slightly. Lack of confidence ranked highest at 9/36 (25.0%), followed by limited understanding of other specialties at 8/36 (22.2%). Three challenges, including limited clinical experience, difficulty in explaining complex nuclear medicine concepts, and time constraints, were each reported by 5/36 (13.9%). The least frequently mentioned challenges in this cohort were insufficient involvement in case discussions (4/36, 11.1%) and lack of familiarity with the cases being discussed (3/36, 8.3%). The differences between groups were not statistically significant (p-value = 0.1). Table 4 summarizes these results.

**Table 4:** Challenges

Challenges	n (%) among 73 responses in all cohort	n (%) among 36 responses received from non- nuclear medicine experts	p-value
Limited understanding of other specialties	24/73 (32.9%)	8/36 (22.2%)	0.251
Time constraints or scheduling conflicts	22/73 (30.1%)	5/36 (13.9%)	0.065
Limited clinical experience	19/73 (26.0%)	5/36 (13.9%)	0.150
Lack of confidence in presenting or participating	16/73 (21.9%)	9/36 (25.0%)	0.719
Insufficient involvement in case discussions	12/73 (16.4%)	4/36 (11.1%)	0.460
Difficulty in explaining complex nuclear medicine concepts	10/73 (13.7%)	5/36 (13.9%)	1.00
Lack of familiarity with the cases being presented	10/73 (13.7%)	c (8.3%)	0.539

Suggestions for Improvement

Respondents offered several suggestions to enhance the impact of nuclear medicine trainees in MCCs. Key recommendations included increasing mentorship and guidance from faculty members (41/73, 56.2%), improving communication training specific to multidisciplinary teams (34/73, 46.6%), and encouraging more active participation in case discussions (29/73, 39.7%). Other suggestions included increasing exposure to cases outside the nuclear medicine domain (27/73, 37.0%), providing more structured learning opportunities (16/73, 21.9%), and fostering collaborative activities outside of rounds (13/73, 17.8%).

There was some variability among non-nuclear medicine specialists, although it was not statistically significant (p-value = 0.493). This group placed more emphasis on encouraging active participation in case discussions (19/36, 52.8%), followed by increasing mentorship (15/36, 41.7%), with the least emphasis on structured learning opportunities (6/36, 16.7%). Table 5 summarizes these suggestions.

**Table 5:** Suggestions

Suggestions for enhancing the impact	n (%) among 73 responses in all cohort	n (%) among 36 responses received from non- nuclear medicine experts	p-value
Increase mentorship or guidance from faculty members	41/73 (56.2%)	15/36 (41.7%)	0.154
Improve communication training specific to multidisciplinary teams	34/73 (46.6%)	8/36 (22.2%)	0.014
Encourage active participation in case discussions	29/73 (39.7%)	19/36 (52.8%)	0.197
Increase exposure to other specialties	27/73 (37.0%)	8/36 (23.1%)	0.120
Provide more structured learning opportunities for trainees	16/73 (21.9%)	6/36 (16.7%)	0.521
Foster collaborative activities outside of rounds	13/73 (17.8%)	10/36 (27.8%)	0.230

Feedback From Open-Ended Questions

In addition to the quantitative responses, the open-ended questions provided valuable insights into the perceived benefits and challenges of nuclear medicine trainees' involvement in MCCs. Out of 31 open-ended responses, 0.51% (n = 16), equal to 22% of the total sample population and 44% of the non-nuclear medicine specialist group, appreciated the contributions of nuclear medicine trainees, particularly in complex cases that require advanced imaging techniques.

Non-nuclear medicine specialists overwhelmingly appreciated the contributions of nuclear medicine trainees, particularly in complex cases that require advanced imaging techniques. Respondents highlighted the value of trainees’ expertise in hepatopancreatobiliary (HPB) conditions and neuroendocrine tumors, where their up-to-date knowledge on imaging modalities such as PET was seen as enriching discussions and improving diagnostic accuracy. One participant noted, "The trainees’ insights into nuclear imaging in neuroendocrine cancers helped clarify treatment plans, especially in the context of advanced imaging techniques."

However, participants also pointed to areas where trainee involvement could be optimized. One concern raised was the inconsistent attendance of nuclear medicine trainees at MCCs. Respondents noted that while trainees add valuable input, their absence from certain rounds, especially those where nuclear medicine imaging is crucial, diminishes their impact. One non-nuclear medicine specialist remarked, "It would be helpful if trainees could attend all rounds related to imaging, as their absence from key rounds reduces the value of their participation."

Additionally, technical challenges were mentioned, particularly in virtual rounds, where issues with screen sharing and presentation of imaging were cited as occasional barriers to effective communication. Respondents suggested that improved technical support would help facilitate smoother participation by trainees.

From the perspective of nuclear medicine participants, there was a call for better preparation and more active participation from trainees in case discussions. Several respondents emphasized that trainees should come prepared with a deeper understanding of the cases they are presenting, which would allow them to contribute more meaningfully. One nuclear medicine trainee shared, "Sometimes I feel like I lack enough background knowledge of the other specialties involved, which hinders my ability to fully engage in the discussions."

Finally, there were concerns about the workload, particularly for new trainees, who may find the volume of cases and the early timing of rounds overwhelming. Suggestions to address this included reducing the workload for junior trainees and providing more structured case preparation to alleviate stress. "Having a lighter workload in the early stages of training would be beneficial," one participant commented.

## Discussion

Educational impact

The variety of cancer conferences at tertiary educational centers plays a central role in addressing challenges related to decision-making and treatment planning for cancer patients. These multidisciplinary case conferences involve all branches of medicine, including diagnostic imaging, laboratory, clinical, and surgical departments. However, MCCs require significant resources and time from medical staff, which limits the ability of the trainees to actively participate. Despite this, MCCs provide valuable educational opportunities for trainees, not only for learning directly related to their specialty fields but also for enhancing interpersonal skills and decision-making abilities [1-6,8-10,12,13].

The introduction of a dedicated MCC rotation for nuclear medicine trainees, with continuous active case participation, has been well-received and highlights the structured design of our program. This aligns with Ogut et al., who demonstrated that specialized study modules (SSM) in cross-sectional anatomy improve engagement and learning. Ensuring consistent trainee attendance in MCCs parallels the SSM framework, in which deliberate scheduling and protected time enhance teamwork and learning outcomes [14].

Our study demonstrates the significant role nuclear medicine trainees play in enhancing both the educational experience and collaborative atmosphere of MCCs. The overwhelmingly positive feedback on the impact of trainees on interprofessional collaboration (78.1%) and clinical knowledge (83.6%) underscores a consistent perception across specialties that nuclear medicine trainees are particularly valuable in complex cases requiring advanced imaging, particularly in cases requiring advanced imaging interpretation, such as neuroendocrine tumors and hepatopancreatobiliary conditions.

Influence on patient outcomes

The results also reveal that nuclear medicine trainees contribute to improved patient outcomes, with over 56/73 (76.7%) of respondents believing their involvement positively influences the management of cancer patients. Across specialties, trainees were consistently perceived as enhancing the effectiveness of treatment planning, particularly in cases requiring advanced imaging interpretation.

These findings are consistent with prior studies suggesting a collaborative, multidisciplinary approach improves patient outcomes. Neri et al. reported that radiologist attendance could change diagnostic strategy or refine therapeutic decisions in 25-50% of cases discussed [1]. Similarly, the American Society of Clinical Oncology (ASCO) survey showed that MTB discussions led to changes in treatment plans for 44-49% of breast cancer patients and 47-50% of colorectal cancer patients, with changes ranging from 1% to 25% [2]. These literature comparisons support our findings, highlighting that the contributions of nuclear medicine trainees parallel those observed for radiologists and internal medicine residents, confirming their value in multidisciplinary care.

Barriers and challenges

While the overall response was positive, several challenges were identified that can impact the effectiveness of trainee participation. Inconsistent attendance was the most frequently cited issue, reducing the benefits trainees provide in key discussions. Limited clinical experience, time constraints, and lack of familiarity with other specialties were also noted. Addressing these barriers could involve structured rotations, mentorship programs, and increased exposure to interdisciplinary cases.

Previous studies have quantified the increased workload for radiologists in MDTMs over a 15-month period, showing substantial additional burden and limited time for other responsibilities [12]. Our findings suggest similar workload concerns for nuclear medicine trainees, emphasizing the need for protected time for case preparation and meeting participation.

Technical considerations

Technical issues in virtual rounds, including screen-sharing problems and difficulties presenting nuclear medicine imaging, point to the need for improved logistical support. Enhancing technical infrastructure would enable clearer communication of imaging expertise and more effective trainee participation.

Previous studies have briefly mentioned infrastructure challenges [2,4,6,8-10,12], but their importance may be underestimated, particularly for radiologists. Surveys indicate that 55% of radiologists need high-resolution image projection and 32.7% require Picture Archiving and Communications Systems (PACS) workstations [1]. Properly equipped rooms have also been identified as key for establishing effective MTBs [13].

Trainee feedback and educational support

The feedback from nuclear medicine trainees emphasized the need for better preparation and a more structured approach to learning. Ensuring that trainees are adequately prepared for the cases they will be discussing will allow them to participate more meaningfully, which could be supported through targeted educational interventions and case preparation materials provided prior to rounds.

Individual learning styles may also influence how trainees engage with and benefit from these sessions. For example, visual learners may benefit from diagrams or imaging examples, auditory learners from discussions and explanations, and kinesthetic learners from hands-on or interactive activities. Recognizing these differences could improve motivation and learning outcomes, highlighting the need for diverse instructional strategies [15].

The feedback concerning workload further suggests that the early stages of nuclear medicine training could be better supported by reducing case volume or providing structured support to alleviate stress. This challenge aligns with previous studies, which identified limited preparation time as a significant barrier for radiologists, with 46.6% reporting that heavy clinical workloads restricted their ability to thoroughly review cases. Other obstacles included insufficient staffing, scheduling conflicts, and a lack of recognition of the additional workload associated with participation in MTBs [1].

Study limitations

This study prospectively evaluated nuclear medicine trainees with defined responsibilities and structured schedules. Limitations include the cross-sectional survey design, potential response bias, and small sample size. Additionally, the voluntary nature of participation may overrepresent respondents with strong opinions, and the absence of longitudinal follow-up limits assessment of sustained impacts over time. The single-center design also limits generalizability.

## Conclusions

In summary, nuclear medicine trainees are invaluable assets to MCCs, contributing to both patient care and the learning environment. Optimizing their participation through enhanced mentorship, structured learning opportunities, and improved logistics can maximize their impact and benefit both trainees and patients. Addressing inconsistent attendance, time constraints, limited clinical experience, and technical difficulties will strengthen the multidisciplinary role of nuclear medicine trainees.

## Appendices

The questionnaire used for this study

Evaluating the Impact of Nuclear Medicine Trainees in Multidisciplinary Rounds (2)

Thank you for participating in this survey. The goal of this study is to assess the role of nuclear medicine trainees in fostering interprofessional collaboration during multidisciplinary rounds. Your feedback will provide valuable insights into how their involvement can enhance collaborative practices in both.

*Required educational and clinical settings. This questionnaire is provided to attendees of all MCCs separately. If you have already completed it, you may skip filling it out again. Please be assured that all responses will remain confidential.

1. What is your professional role? (Please select one)

☐ Nuclear Medicine Trainee

☐ Nuclear Medicine Specialist

☐ Radiologist

☐ Thoracic Surgeon

☐ General Surgeon/Colorectal Surgeon

☐ Urologist

☐ Medical Oncologist

☐ Radiation Oncologist

☐ Anatomical Pathologist

☐ Hematopathologist

☐ Hematologist

☐ Endocrinologist

☐ Otolaryngologist/Head and Neck Surgeon

☐ Gastroenterologist

☐ Gynecologic Oncologist

☐ Pediatrician/Subspecialist in Pediatrics

☐ Nuclear Medicine Technologist

☐ Nurse

☐ Administrative Staff (e.g., Secretary/Organizer)

☐ Trainee (Medical Student, Non-Nuclear Medicine Resident/Fellow)

2. Which multidisciplinary round do you evaluate the role of Nuclear Medicine trainees in? (Please select from the following list.)

☐ GI Round

☐ GU Round

☐ HPB Round

☐ Breast Round

☐ Pediatric Round (CHEO)

☐ Thoracic Round

☐ Melanoma Round

☐ Lymphoma/ PET Round

☐ Foregut Round

☐ Endocrine Round

3. How many years of professional experience do you have in your current role?

☐ 0-1 years

☐ 2-5 years

☐ 6-10 years

☐ 11+ years

4.  How frequently do you participate in multidisciplinary rounds? (Please select one)

☐ Daily

☐ Weekly

☐ Monthly

☐ Rarely

☐ Never

5. What is your primary role during multidisciplinary rounds? (Please select one)

☐ Nuclear Medicine Specialist reviewing images in the round

☐ Nuclear Medicine Trainee reviewing images on behalf of the attending physician

☐ Physician who refers cases to the round

☐ Physician contributing to discussions and treatment planning

☐ Coordinating referrals, bookings, and treatments

☐ Observing/Listening

6. Impact assessment

In your opinion, how impactful is the participation of nuclear medicine trainees in multidisciplinary rounds on interprofessional collaboration? (Please select one)

☐ Very impactful

☐ Somewhat impactful

☐ Neutral

7. What specific contributions do you believe nuclear medicine trainees bring to multidisciplinary rounds? (Please select all that apply)

☐ Enhanced understanding of nuclear medicine imaging

☐ Improved patient management strategies

☐ Fresh perspectives on cases

☐ Enhanced communication between disciplines

☐ Accelerated management by facilitating further referrals/imaging

☐ Better understanding of complex imaging techniques

☐ Improved collaboration with other specialties

☐ Strengthened interaction between trainees and faculty

☐ Support for professional development

☐ Bridging the gap between theory and practice

☐ Improved team dynamics

8. Outcome assessment

How do you perceive the involvement of nuclear medicine trainees in multidisciplinary rounds?

☐ Significantly improves outcomes

☐ Moderately improves outcomes

☐ No effect on outcomes

9. How do multidisciplinary rounds contribute to the education of nuclear medicine trainees? (Please select all that apply)

☐ Provides real-time learning opportunities

☐ Encourages critical thinking

☐ Fosters communication skills

☐ Enhances teamwork abilities

☐ Builds confidence

☐ Facilitates active learning for trainees

☐ Provides engagement with faculty members

☐ Offers opportunities to receive critical feedback

10. Effectiveness of participation

Please rate the effectiveness of nuclear medicine trainees' participation in multidisciplinary rounds in enhancing your clinical skills and knowledge. (Please select one)

☐ Very effective

☐ Effective

☐ Neutral

11. What challenges have you encountered regarding the participation of nuclear medicine trainees in multidisciplinary rounds?(Please select all that apply)

☐ Limited clinical experience

☐ Limited understanding of other specialties

☐ Difficulty in explaining complex nuclear medicine concepts

☐ Time constraints or scheduling conflicts

☐ Insufficient involvement in case discussions

☐ Lack of confidence in presenting or participating

☐ Lack of familiarity with the cases being presented

12. What suggestions do you have for enhancing the impact of nuclear medicine trainees in multidisciplinary rounds? (Please select all that apply)

☐ Provide more structured learning opportunities for trainees

☐ Encourage active participation in case discussions

☐ Increase mentorship or guidance from faculty members

☐ Improve communication training specific to multidisciplinary teams

☐ Foster collaborative activities outside of rounds (e.g., joint case studies)

☐ Increase exposure to other specialties

13. Any additional comments or suggestions regarding the improvement of nuclear medicine trainees' participation in MCC rounds? (free text response)
